# Global network of computational biology communities: ISCB's Regional Student Groups breaking barriers

**DOI:** 10.12688/f1000research.20408.1

**Published:** 2019-09-02

**Authors:** Sayane Shome, R. Gonzalo Parra, Nazeefa Fatima, Alexander Miguel Monzon, Bart Cuypers, Yumna Moosa, Nilson Da Rocha Coimbra, Juliana Assis, Carla Giner-Delgado, Handan Melike Dönertaş, Yesid Cuesta-Astroz, Geetha Saarunya, Imane Allali, Shruti Gupta, Ambuj Srivastava, Manisha Kalsan, Catalina Valdivia, Gabriel J. Olguin-Orellana, Sofia Papadimitriou, Daniele Parisi, Nikolaj Pagh Kristensen, Leonor Rib, Marouen Ben Guebila, Eugen Bauer, Gaia Zaffaroni, Amel Bekkar, Efejiro Ashano, Lisanna Paladin, Marco Necci, Nicolás N. Moreyra, Martin Rydén, Jordan Villalobos-Solís, Nikolaos Papadopoulos, Candice Rafael, Tülay Karakulak, Yasin Kaya, Yvonne Gladbach, Sandeep Kumar Dhanda, Nikolina Šoštarić, Aishwarya Alex, Dan DeBlasio, Farzana Rahman

**Affiliations:** 1 Bioinformatics and Computational Biology Program, Iowa State University, Iowa, USA; 2 Genome Biology Unit, European Molecular Biology Laboratory (EMBL), Heidelberg, Germany; 3 Science for Science for Life Laboratory, Department of Immunology, Genetics and Pathology, Uppsala University, Upsala, Sweden; 4 Department of Biomedical Sciences, University of Padova, Padova, Italy; 5 Department of Biomedical Sciences, Institute of Tropical Medicine, Antwerp, Belgium; 6 Department of Mathematics and Computer Science, University of Antwerp, Antwerp, Belgium; 7 KZN Research and Innovation Sequencing Platform, University of KwaZulu Natal, Durban, South Africa; 8 Graduate Program in Bioinformatics, Universidade Federal de Minas Gerais, Belo Horizonte, Minas Gerais, Brazil; 9 Institut de Biotecnologia i de Biomedicina, Universitat Autònoma de Barcelona, Bellaterra, Spain; 10 European Molecular Biology Laboratory, European Bioinformatics Institute, Wellcome Genome Campus, Hinxton, UK; 11 School of Microbiology, Universidad de Antioquía, Medellín, Colombia; 12 Colombian Tropical Medicine Institute (ICMT), Universidad CES, Medellín, Colombia; 13 Department of Biological Sciences, University of South Carolina, South Caroli a, USA; 14 Department of Biology, Faculty of Sciences, Mohammed V University in Rabat, Rabat, Morocco; 15 Division of Computational Biology, Department of Biomedical Sciences, Institute of Infectious Disease and Molecular Medicine, Faculty of Health Sciences, University of Cape Town, Cape Town, South Africa; 16 School of Computational and Integrative Sciences, Jawaharlal Nehru University, New Delhi, India; 17 Department of Biotechnology, Indian Institute of Technology Madras, Chennai, India; 18 Ecosystem’s Health Laboratory, Universidad Andres, Bello Santiago, Chile; 19 Center for Bioinformatics, Simulations and Modelling, Universidad de Talca, Talca, Chile; 20 Interuniversity Institute of Bioinformatics in Brussels, Université libre de Bruxelles-Vrije Universiteit Brussel, Brussels, Belgium; 21 ESAT-STADIUS KU Leuven, Heverlee, Heverlee, Belgium; 22 DTU Health Technology, Technical University of Denmark, Lyngby, Denmark; 23 The Bioinformatics Center, Biology and Biotech Research and Innovation Center, University of Copenhagen, Copenhagen, Denmark; 24 Luxembourg Centre for Systems Biomedicine (LCSB), University of Luxembourg, Esch-sur-Alzette, Luxembourg; 25 Swiss Institute of Bioinformatics (SIB), University of Lausanne, Lausanne, Switzerland; 26 Molecular Diagnostics, Laboratory Services, APIN Public Health Initiatives, Abuja, Nigeria; 27 Genetics and Evolution of Buenos Aires (IEGEBA), CONICET-UBA, Institute of Ecology, Buenos Aires, Argentina; 28 Biomedical Centre, Faculty of Medicine, Lund University, Lund, Sweden; 29 Laboratorio de Biotenología de Plantas, Universidad Nacional de Costa Rica (UNA), Heredia, Costa Rica; 30 Quantitative and Computational Biology, Max Planck Institute for Biophysical Chemistry, Göttingen, Germany; 31 Research Unit for Bioinformatics, Rhodes University, Grahamstown, South Africa; 32 Izmir Biomedicine and Genome Center, Dokuz Eylül University, Izmir, Turkey; 33 Hacettepe University, Faculty of Science, Department of Biology, Ankara, Turkey; 34 University Medical Center Rostock, University Heidelberg, Heidelberg, Germany; 35 La Jolla Institute for Allergy and Immunology, La Jolla Institute for Immunology, California, USA; 36 Centre of Microbial and Plant Genetics, KU Leuven, Leuven, Belgium; 37 Roche Diagnostics Automation Solutions GmbH, Roche, Waiblingen, Germany; 38 Computational Biology Department, Carnegie Mellon University, Pittsburgh, USA; 39 Genomics and Computational Biology Research Group, University of South Wales, Pontypridd, UK; 40 School of Human and Life Sciences, Canterbury Christ Church University, Kent, UK

**Keywords:** Student organizations, Symposia, Bioinformatics, Computational Biology, Workshops, Education, Virtual seminars, ISCB Student Council, Regional Student Groups, ISCB, early career bioinformaticians, collaboration, networking

## Abstract

Regional Student Groups (RSGs) of the International Society for Computational Biology Student Council (ISCB-SC) have been instrumental to connect computational biologists globally and to create more awareness about bioinformatics education. This article highlights the initiatives carried out by the RSGs both nationally and internationally to strengthen the present and future of the bioinformatics community. Moreover, we discuss the future directions the organization will take and the challenges to advance further in the ISCB-SC main mission: “Nurture the new generation of computational biologists”.

## Introduction

Regional Student Groups (RSGs) are student-oriented groups affiliated to the Student Council of the International Society of Computational Biology (ISCB-SC) ^1^. Aligned with the mission and objectives of the parent organization ISCB-SC, RSGs were formulated to promote networking amongst budding computational biologists in the local geographical regions. Since its formation in 2006 with four RSGs (Netherlands, India, Korea, and Singapore), the program has come a long way with 30 active RSGs operating around the world, together constituting a global network of over 2000 members. RSGs are completely autonomous and over the last decade, with the economic support of the ISCB-SC, have organized a large variety of activities according to their community needs.

Computational biology and bioinformatics are relatively new and multidisciplinary areas and hence undergraduate education on these topics is scarce. Instead, young researchers in these fields often come from other disciplines such as molecular biology, computer science or physics and need to complement their background education with knowledge from other disciplines. Additionally, the growing importance of computational biology in a wide range of biological fields is also motivating young researchers in pure experimental groups to get more expertise about this emerging field ^2^. For all these reasons, the RSGs are playing an instrumental role in promoting networking and knowledge transfer related to computational biology topics amongst student researchers. These are typically achieved by organizing offline and online networking and educational events such as workshops, symposiums, hackathons, online competitions, virtual seminars, and many others.

When the ISCB-SC was formulated in 2004, one of its main challenges was due to students from different geographical regions having different needs that could hardly be addressed by a single activity or event. As a consequence, the RSG-program was created in 2006 so people living in specific regions could articulate their own activities that will, in turn, enhance networking and the emergence of regional leaders that will later be potential successors to the ISCB-SC leadership.

Each RSG has a different organizational architecture depending on the local requirements, objectives and initial set up. For instance, some RSGs have been built from scratch, whereas some others have been created in collaboration with existing student organizations such as COMBINE (Australia) and SASBi (South African Student Bioinformatics Society). Irrespective of the organizational setup, there are a few requirements which have been kept mandatory for setting up an RSG at the region. The steering team requires a President and a Secretary who are primarily students, and a faculty advisor who is a member of the ISCB. Also, it is highly encouraged that this steering team has a representation from multiple universities/institutes to promote local collaborations in the field. Setting up an RSG involves taking into consideration operational and logistics aspects which can be a challenge at the start. However, this is a great learning experience for the involved young researchers as they can develop transferable soft skills such as conferences and symposia organization, fundraising, conflict management, team building, project executions and many others. Some of the operational hurdles and related case-studies have been highlighted in our previous article ^3^.

All 30 ISCB-SC RSGs have organized a diverse battery of events tailored to address specific needs and requirements from their target audiences and members. We will discuss some of the many successful ventures carried out by the different RSGs

## Regional events are great networking platforms

Since 2005, the ISCB-SC organizes several events at distinct levels of national or international cooperation. One-day symposia, namely SCS ^4, 5^, ESCS ^6^, LA-SCS ^7^ and SCS Africa ^8^, are yearly organized as satellite events to the main ISCB official conferences: ISMB, ECCB, ISCB-LA and ISCB-Africa respectively. These symposia constitute the ISCB-SC flagship events and have itinerant locations for each edition.

Although these events constitute the perfect occasion where the general ISCB-SC leadership can get together and discuss in person the plans and balances for the current year, travel costs and accommodation make it difficult for all students to attend, venues are often located at capital cities and the official language is English which can intimidate and make it difficult for young students to participate.

To leverage these obstacles and inspired by the success of the aforementioned symposia, various RSGs have organized one-day symposia either under the aegis of a major conference or as a stand-alone event. In the next section, we revisit the highlights of some of the latest RSG organized events, such as symposia, workshops and social meetings, grouped by continent.

### Latin America

Since 2016, RSG-Brazil has successfully organized three symposia editions in collaboration with the Brazilian Association of Bioinformaticians and Computational Biologists (AB3C), with an average of 70 delegates per year.

RSG-Colombia organized two regional meetings in Medellin and Bogotá in 2017. Their national meeting is held every two years during the biennial Colombian Congress in Bioinformatics ( http://ccbcol.org/) in collaboration with the Colombian Society of Bioinformatics and Computational Biology ( http://www.sc2b2.org/). The first edition of this national meeting was held in Cali, which experienced an intense day of science and networking, with 12 student talks from 8 Colombian universities.

Similar to previous SAJIB versions, the 3rd Argentine Symposium of Young Bioinformatics Researchers (SAJIB) was held on July 28–29 2018 at Fundación Instituto Leloir (FIL), in Buenos Aires, Argentina. SAJIB ^9^ was carried out for a period of two days; one day reserved for workshops and another day for the symposium. In this edition, they offered two courses: “Introduction to Python” and “Filtering, assembly, and assessment from NGS data.” Forty-five students and young researchers attended both. The second day included the symposium which gathered 34 people. Additionally, RSG Argentina has been working, joining efforts to expand the bioinformatics community over the country.

During 2017–2018, RSG-Colombia has been involved in the organization and teaching of different bioinformatics courses such as tutorial sessions on metagenomic data analysis at Universidad de Antioquia and Universidad del Valle. A similar approach has been employed by RSG-Chile for organizing tutorial workshops on topics of genomics, coarse-grained Molecular Dynamics, R programming etc. Recently initiated in 2017, RSG-Chile primarily has the presence in two campuses in Chile. They have been working on spreading it to other universities. Despite various logistic and technical hurdles, they have successfully managed two workshops in the past year and have received a good response from the audience. Technical-oriented seminars have also been organized by RSG-Brazil such as “Genomic data analysis using Python programming” and a hands-on course on bioinformatic analysis of data related to tropical diseases.

### Europe

RSG-Spain has organized several Bioinformatics Student Symposium editions, either preceding the Spanish Bioinformatics Symposium or as standalone events, typically gathering over 50 attendees for selected scientific and/or career talks, talks, workshops and networking activities.

RSG-UK has organized several bioinformatics and life science student symposia since its inception along with several workshop and seminars gathering UK-wide community of bioinformatics students and scientists ^10^.

In 2018, RSG-Turkey organized a one-day symposium as a satellite meeting to the most prominent international bioinformatics meeting in Turkey, 4th International Symposium on Health Informatics and Bioinformatics (HIBIT).

Collaborations between various RSGs have resulted in successful events such as the BeNeLux Bioinformatics Conference (which was organized jointly by RSG-Belgium, RSG-Netherlands, and RSG-Luxembourg).

Apart from formal sessions, informal setups also have proved to be good networking events well-received by youth computational biologists across Europe. In 2017, RSG-Luxembourg organized a series of science pub quiz events named ‘Sci-Pub,’ where scientists and citizens casually met to answer fun questions about science and win prizes. The invitations were extended to the broad public through Facebook adds as well as students from the University of Luxembourg from various backgrounds. After the end of each session, the assessment of knowledge was performed through written questionnaires. In total, for “Sci-Pub” events spanned the second semester of 2017 with an average attendance of 30 people per session.

Similarly, one of the recurring activities of RSG-Switzerland called “Bioinformatics in the Pub” has been very much appreciated by the Swiss students. It is a monthly meeting that gathers bioinformaticians from different departments of the University of Lausanne (UNIL) and the polytechnic school EPFL in a friendly atmosphere. This event is planned to be extended in the future to Basel and Zurich. RSG-Switzerland is also closely related to the Swiss Institute of Bioinformatics (SIB). Encouraged by the SIB, RSG-Switzerland aims to enable students to discover other careers paths outside of academia. It is in this spirit; they were given the opportunity to organize a full session during the Basel Computational Biology conference BC2 named “Entrepreneurs’ stories: opportunities and challenges of starting your own company.–

RSG-Denmark focused on smaller events that facilitate networking between computational biologists around Copenhagen. They have organized workshop events and a regular bioinformatics coffee meetup. At CBio Coffee events students got a chance to ask questions on how to further their career for example “what elective courses should I focus on?”, “how can a foreign expat best further his/her career in Denmark?” or “How would it be to work in a specific company as a data scientist/bioinformatician?” On the other hand, their young and neighbouring community RSG-Sweden organized career events that have been much appreciated. Besides, RSG-Sweden community have organized journal several clubs and online networking hours.

### Africa

RSG-South Africa organized a one-day student Symposium in association with the South African Bioinformatics (SASBi) and Genetics (SAGS) Societies ^11^ for two consecutive years.

Besides, to encourage student presenters to showcase their research work, RSGs also invited eminent speakers who are distinguished scientists in their area. RSG-Northern Africa organized a conference on “Personalized Medicine” and invited Prof. Peter Tonellato from Harvard Medical School as their keynote speaker in 2015.

RSG-South Africa organized a session of exhibitions and workshops at the National Science Festival, with prime focus at school-going scientists.

Irrespective of venue locations, events organized by RSGs have helped to facilitate interactions from students of other countries involved as well. For instance, most of the events hosted by RSG-Northern Africa 2013 generally have venues in different parts of Morocco. But, students of other neighbouring countries such as Tunisia and Mali could also participate in RSG-Northern Africa symposium as they were provided with travel fellowships to travel to the event venue in Nador, Morocco. In 2017, the 2nd RSG-Northern Africa Symposium was organized in December in Casablanca, Morocco. This event was co-founded by H3AbioNet that supported three travel fellowships for students coming from Tunisia and Mali. This was followed by a further meeting and student symposium alongside the International Society for Computational Biology (ISCB) and the African Society for Bioinformatics and Computational Biology’s (ASBCB) biennial conference in October of 2017 in Entebbe, Uganda. Forty-four students from 9 different countries across the continent attended including participants from various levels of expertise. The symposium featured a keynote speaker, Dr Segun Fatumo, as well as student presentations and a poster session. These events are valuable for students in terms of experience as well as for networking within the continent not only the country ^12^.

### USA

Some symposia have also extended to more than a one-day schedule and comprised various events. In December 2017, RSG-Southeastern USA organized their first research symposium for 2017–2018 in collaboration with University of South Carolina; University of South Florida, St. Petersburg; and University of Alabama, Birmingham. There were research talks from professors from across these universities, hands-on workshops on machine learning, designing pipelines for genetic analyses and three-dimensional modelling of biomolecules. Undergraduate and graduate students also had the opportunity to give talks and present posters at the symposium.

For students who search for initiatives where they can acquire hands-on experience with new concepts and techniques, stand-alone workshops typically appeal a lot. RSG-District of Columbia (USA) has been organizing summer workshops on “Bioinformatics, Genomics and Computational Biology” at the University of Maryland campus, where they have targeted to involve researchers from different programming backgrounds to benefit from the workshop ( https://iscb-dc-rsg.github.io/workshop2017/).

### Asia

For panel discussion at “Career Opportunities in Computational Biology and Bioinformatics” held during InCoB 2018 (International Conference of Bioinformatics), RSG-India invited various scientists and professionals who have considerable experience in their respective fields of academia and industry in India and abroad. The event held at Jawaharlal Nehru University, New Delhi focussed on the discussion about different career opportunities and skill sets required for a job profile in academia or industry.

## Online platform for networking and knowledge transfer

The increasing number of social media platforms and usage of web resources have led to new communication channels for networking. The community of STEM researchers is expanding its presence on Twitter and other social media platforms to voice their opinions on crucial matters, share their research work, and promote science. Social media platforms allow people to get connected quickly and frequently irrespective of their locations. The majority of RSGs interact with their members via Twitter, curated mailing lists, online groups, and official Facebook pages.

Several RSGs are spreading their branches by launching online initiatives. The webinar project started a few years ago by the RSG-Turkey has reached the audience of more than 350 people ( https://www.bigmarker.com/communities/bioinfonet) in over 30 countries and continues to grow. The RSG-Turkey team has also initiated collaborative sessions with RSG-Colombia and RSG-Denmark. The primary goal of these webinars is to encourage researchers and mainly students to know more about computational biology in their countries as well as abroad. In collaboration with RSG-Turkey, the RSG-Colombia is inviting bioinformaticians and computational biologists working in Colombian universities as speakers. This is an essential step towards increasing the visibility of research work being carried out. Starting in 2019, RSG-Southeastern USA is also organizing online podcasts and talks to engage, foster and increase participation amongst the Bioinformatics student groups across Southeastern USA region.

Online platforms also benefit regions with limited access to resources, for instance in the case of RSG-Western Africa. H3ABioNet, a Pan African Bioinformatics network comprising 32 Bioinformatics research groups distributed amongst 15 African countries with two partner institutions in the USA, is a major supporting network behind RSG-Western Africa. RSG Western Africa has primarily benefited from the H3ABioNet as they provide free content through webinars, funding tailored for students in resource-limited countries to attend career conferences and workshops and more recently, facilitating participation Bioinformatics development by inclusion in a variety of H3ABioNet projects.

In addition to a webinar series, online competitions have also organized programming challenges ’CASPita’, by RSG-Italy, and ‘Research writing competition,’ by RSG-India. RSG-Italy organized a programming challenge inspired by the CASP (Critical Assessment of Structure Prediction) ^10^ competition and hence named it ‘CASPita.’ The participants were challenged to write a parser for text output of BLAST. A few groups for all over Italy joined the competition and were evaluated by coding skill and biological accessibility. The winner was awarded a monetary prize funded by the ISCB.

In addition, a “Scientific Writing Competition” was organized by RSG-India at the end of 2018. The participants were asked to submit an essay entry in any of the three different topics provided. The topics were selected to encourage students to be creative and innovative while requiring a prerequisite knowledge of computational biology and bioinformatics and being up-to-date with the latest advancements and present status of the research area. The competition invited commendable participation from students from across the country and the best entries, graded by creativity, innovation, futuristic outlook and other aspects, were awarded.

## Future directions and plans: Opportunities and obstacles

At present, the RSG program is extending further to new regions such as Lebanon, Czech Republic, Bangladesh, and other countries. Existing RSGs are attempting to expand further in their areas of operations; such as RSG-Spain, which has the intention to split into several local nodes to better cover the large region. Similar efforts have been carried out by RSG-Australia to have different divisions across the country. Many new RSGs started in the past three years such as RSG-Costa Rica, Colombia, Chile, Greece, Bangladesh, Jordan, and Southeastern-USA.

The relatively new RSG-Sweden has also established itself as a bridging entity between the student community and industry in the field of computational biology in Sweden since their recent initiation in 2018. To further expand the community and make it inclusive, they have established the concept of branches starting with Lund and Stockholm and most recently Uppsala and Gothenburg. In the future, RSG-Sweden aims to collaborate with other RSGs across Europe to be able to share knowledge and ideas and strengthen the global community of the ISCB and its Student Council. Future plans include recruiting new members to the committee and branches and organizing seminars and hackathons.

Currently, RSG Germany is also in the process of re-establishing its connections to students by initiating monthly literature review events and in the planning process of organizing a student symposium in Heidelberg in 2019. Furthermore, the RSG plans to expand its network with German universities and arrange lunch meetings between interested participants along the lines of the Connect movement ( https://connected.mit.edu/about/connector/mit).

RSG Chile has closely collaborated with biotechnologist leaders from all over Latin America who make up the Allbiotech Community. Allbiotech’s purpose is to establish and promote a Latin American community ecosystem that includes all segments of the economy. This collaboration also resulted in the starting up of RSG-Costa Rica. Their focus is to unify the interested community in both disciplines through meetings, workshops and diffusion activities like the ones developed at the ISCB LA-SCS 2018 and Allbiotech 2018, and work together to be a part of the organization team for Allbiotech 2019 that will take place in Costa Rica. Although, new RSGs show zeal and enthusiasm to expand their ventures in the regions, they also face issues with the establishment or team transitions. For instance, to promote networking among the students and postdocs on the west coast, RSGs based in California and Nevada region of the United States was initiated in 2016. They had initial hurdles of expanding their membership, even though they tried by being one of the exhibitors at NCCB (North California Computational Biology) symposium 2016. Later, RSG-California+Nevada was merged with the undergraduate student organization of the University of California, San Diego (UCSD) bioinformatics group for further development.

The way to success and growth when running an RSG is full of challenges. Irrespective of several operational hurdles and obstacles ^13^, the RSGs have been putting efforts to expand the spirit of enthusiasm for computational biology research. In the future, the RSG program aims to expand to new regions, particularly in developing nations, promote collaborations between RSGs and also exploit virtual space via virtual seminar series programs.

## Data availability

No data is associated with this article.
